# Exposure to secondhand smoke and voluntary adoption of smoke-free home and car rules among non-smoking South African adults

**DOI:** 10.1186/1471-2458-14-580

**Published:** 2014-06-10

**Authors:** Olalekan A Ayo-Yusuf, Olubode Olufajo, Israel T Agaku

**Affiliations:** 1 School of Health Systems and Public Health, University of Pretoria, Pretoria, South Africa; 2 Office of the Director, School of Oral Health Sciences, University of Limpopo MEDUNSA campus, Pretoria, South Africa; 3 Department of Epidemiology, Harvard School of Public Health, 677 Huntington Avenue, Boston, MA 02115, USA; 4 Center for Global Tobacco Control, Department of Social and Behavioral Sciences, Harvard School of Public Health, 677 Huntington Avenue, Boston, MA 02115, USA

**Keywords:** Smoking, Policy, Secondhand smoke, Bans, Cars, Homes, Tobacco, Cigarettes, Smoke-free, Non-smokers

## Abstract

**Background:**

Secondhand smoke (SHS) exposure is a well-established health hazard. To determine the effectiveness of existing smoke-free policies and adoption of smoke-free rules in South Africa, we assessed exposure to SHS from several sources among non-smoking adults during 2010.

**Methods:**

Data were analyzed for 3,094 adults aged ≥16 years who participated in the 2010 South African Social Attitudes Survey. Descriptive statistics and multivariate analyses were used to assess presence of smoke-free rules among all South Africans, and prevalence and correlates of SHS exposure at work, at home, and at hospitality venues among non-smokers.

**Results:**

Overall, 70.6% of all South African adults had 100% smoke-free rules in their private cars, 62.5% in their homes, while 63.9% worked in places with 100% smoke-free policies. Overall, 55.9% of all non-smokers reported exposure to SHS from at least one source (i.e., in the home, workplace or at a hospitality venue). By specific source of exposure, 18.4% reported being exposed to SHS at work, 25.2% at home, 33.4% in a restaurant, and 32.7% at a bar. Presence of work bans on indoor smoking conferred lower likelihood of SHS exposure at work among non-smokers (adjusted odds ratio [aOR] = 0.23; 95% CI: 0.09-0.60). Similarly, smoke-free home rules decreased the odds of being exposed to SHS at home among non-smokers (*a*OR =0.16; 95% CI: 0.09-0.30).

**Conclusion:**

Over half of South African adults reported SHS exposure in the home or at public places such as the workplace and at hospitality venues. This underscores the need for comprehensive smoke-free laws that prohibit smoking in all public indoor areas without exemptions.

## Background

On April 19, 2005, South Africa became a party to the World Health Organization’s (WHO) Framework Convention on Tobacco Control (FCTC) [1]. Under this international Treaty which has been ratified by 175 countries, South Africa has a legal obligation to implement and enforce policies that protect non-smokers from involuntary exposure to tobacco smoke. Article 8 of the WHO FCTC requires parties to make enhanced and sustained efforts to protect nonsmoking children and adults from secondhand smoke (SHS) exposure in “indoor workplaces, public transport, indoor public places, and as appropriate, other public places” [2]. Although South Africa has implemented smoke-free laws in indoor public areas, the laws currently allow designated indoor smoking areas in workplaces and other public places [3,4]. A recent air-quality monitoring study in the country’s capital, suggest these measures are ineffective in protecting non-smokers from involuntary SHS exposure. In designated smoking areas of popular eateries which were assessed in the air-quality monitoring study, measured levels of respirable particulate matter ≤ 2.5 microns in diameter (which are released from burning cigarettes) were over seven-fold higher than the WHO standard of 25 μg/m^3^ set for good air quality [5]. Nonetheless, recent legislative advances have the potential to strengthen smoke-free laws in South Africa. For example, the 2007 Amendment of the Tobacco Products Control Act No 83 of 1993 provided opportunities for more broad scale reductions in SHS exposure among vulnerable groups [3]. The new legislation prohibits smoking in private cars if a child < 12 years is a passenger, as well as smoking within a prescribed distance of the entrance to a public place. The proposed law also regulates smoking in selected outdoor areas, with increased fines for violations of indoor smoke-free laws.

Although the new comprehensive smoke-free law is yet to be implemented and is currently opposed by the industry, evidence indicates that such smoke-free laws have a beneficial effect on the public, particularly considering that there is no safe level of exposure to SHS [6]. Not only do such comprehensive smoke-free laws protect non-smokers from involuntary SHS exposure, they also change social norms and can motivate smokers to quit [6]. In addition, such laws have the potential to raise public awareness about the dangers of tobacco smoke and can influence individuals to become more conscious about their exposure to SHS. In this regard, they may have a ripple effect in influencing individuals to voluntarily adopt smoke-free rules in their private homes, cars, and other micro-environments — areas usually out of the reach of smoke-free laws.

However, strict and consistent enforcement of smoke-free laws is required if continued compliance and population support is to be expected [7,8]. In addition, continuous tobacco surveillance is needed to assess the effectiveness of smoke-free policies so as to provide translational science for improvements and enhancements in policy and practice. To provide an insight into South African adults’ voluntary adoption of smoke-free rules in their private home and cars, and the existence of smoke-free policies in the work environment, as well as exposure of non-smokers to SHS at work, hospitality venues and private homes, this study analyzed nationally representative data of South African Adults from the 2010 South African Social Attitudes Survey (SASAS).

## Methods

### Survey design/sample

This secondary data analysis involved a nationally representative sample of South African adults aged ≥ 16 years who participated in the 2010 (n = 3,094, response rate = 85.8%)wave of the South African Social Attitudes Survey (SASAS). The survey samples were drawn from the master sample of the Human Sciences Research Council (HSRC). The surveys used a multi-stage probability sampling strategy with census enumeration areas as the primary sampling unit and the stratification of the enumeration areas was done by the socio-demographic domains of province, geographical sub-type and the four population groups [9]. This Study was approved by Human Sciences Research Council ethics committee.

#### Socio-demographic characteristics

Socio-demographic characteristics assessed included age (16–24, 25–34, 35–44, 45–54 or ≥ 55 years); educational attainment (<12, = 12, or > 12 years of schooling); sex (male or female); ethnicity (self-identification as Black African, Colored (mixed ancestry), Indian/Asian, or White); marital status (never married, separated/widowed/divorced, or married); and region (urban or rural).

#### Current tobacco smoking, exposure to, and perceptions about SHS

Current tobacco smokers were defined as respondents who reported smoking hand-rolled or manufactured cigarettes, cigars, pipes, or water-pipes daily or on some days. Exposure to SHS at different locations including at home, work, shabeens (i.e., local informal bars), bars, or clubs, as well as at restaurants were assessed separately, with the stem question: ‘In the past 30 days, about how many days would you say you were in a place where someone smoked close (no separation, but in the same area) to you?’ Categorical responses were ‘Never’, ‘1-5 days’, ‘11-15 days’, ‘16-20 days’, or ‘more than 20 days’. All responses other than ‘Never’ were categorized as being exposed to SHS in the respective environments assessed.

Perception about the harmfulness of SHS was assessed using the question ‘In your opinion, to what extent is exposure to second-hand smoke (cigarette smoke from others) harmful to non-smokers health?’ Respondents who answered ‘Very harmful’, or ‘Somewhat harmful’ were categorized as believing that SHS exposure was harmful, whereas a response of ‘Not harmful’ or ‘Do not know/can’t choose’ was categorized to indicate lack of correct knowledge about the harmfulness of SHS exposure.

#### 100% Smoke-free policies/rules

Smoking restrictions at work, in the home and in private cars were assessed separately using the question: ‘Which of the following best describes smoking at your work, home or car?’ Categorical responses were provided separately for each of the three environments (work,home and cars), and were: ‘Smoking is allowed’, ‘Smoking is generally banned with few exceptions’, or ‘Smoking is never allowed’. Respondents who indicated that smoking was never allowed in the respective area assessed were classified as having 100% smoke-free environments, whereas all other responses were categorized as not having complete smoke-free policies without exemptions.

Self-rated importance of 100% smoke-free environments at home, workplaces, hospitals, cafes/restaurants, and at shabeens (informal bars), bars, or clubs were assessed separately and were respectively defined as a report of ‘Very important’ or ‘Somewhat important’ to the question ‘How important is it to you to have 100% smoke-free (no smoking areas) environment in the following places?’

### Analyses

All data were weighted to account for the complex survey design and yield nationally representative estimates. The proportion of adults who had 100% smoke-free policies at work, in their homes, or their cars was calculated overall, as well as by age, education, sex, ethnicity, region and current smoking status. In addition, the proportion of non-smokers who reported being exposed to SHS at work, in their homes, in a café/restaurant or at a shabeen, bar, or club was also assessed overall and further stratified by the afore-mentioned socio-demographic characteristics. Any exposure to SHS was defined as a report by a non-smoker that they were exposed to SHS from at least one of the four environments assessed (i.e., work, home, café/restaurant, or at a shabeen, bar or club).

To assess factors associated with exposure to SHS exposure in the various environments assessed, multivariate logistic regression analyses were performed, adjusting for age, education, sex, ethnicity, and region (*p* < 0.05). All analyses were performed with Stata 11 (StataCorp 2009, College Station, TX).

## Results

### Prevalence of 100% smoke-free policies at home, work, and in private cars among all South African Adults

In total, 18.1% (n = 633) of adults aged ≥ 16 years were current tobacco smokers. Smoking prevalence by ethnicity was as follows: black Africans (13.8%); Coloreds (36.3%); whites (30.8%); and Indians/Asians (22.1%).

During 2010, 62.5% of South African adults had 100% smoke-free policies in their homes, 63.9% worked in places with 100% smoke-free policies, and 70.6% of all adults had 100% smoke-free policies in their private cars. Variations in presence of 100% smoke-free environments were observed among population subgroups (Table 1).

**Table 1:** Proportion of South African Adults aged ≥ 16 years that reported having smoke-free rules at work, at home, and in their private cars, South African Social Attitudes Survey, 2010

**Characteristics**	**Sample**	**Work**	**Home**	**Car**
		**% (95% CI)**	**% (95% CI)**	**% (95% CI)**
		**(n = 2,553)**	**(n = 2,995)**	**(n = 2,654)**
**Overall**	3,112	63.9 (59.5-68.3)	62.5 (60.7-64.4)	70.6 (65.0-76.3)
**Sex**				
Male	1,783	63.2 (61.8-64.6)	61.2 (58.2-64.3)	69.7 (63.0-76.4)
Female	1,311	65.0 (56.9-73.1)	64.5 (62.5-66.5)	72.1 (67.1-77.2)
**Age, years**				
16-24	618	65.3 (62.1-68.5)	61.6 (57.1-66.1)	68.9 (59.3-78.5)
25-34	699	61.8 (53.7-69.9)	63.3 (57.7-68.9)	71.1 (67.3-74.9)
35-44	656	60.4 (53.6-67.2)	60.8 (53.9-67.7)	69.5 (51.4-87.5)
45-54	449	63.9 (57.0-70.8)	66.2 (60.1-72.4)	71.0 (61.4-80.6)
≥55	688	68.9 (60.0-77.8)	62.2 (58.9-65.5)	74.0 (58.1-90.0)
**Education**				
<12 years of schooling	1,683	65.0 (56.9-73.1)	60.6 (56.1-65.0)	71.5 (63.1-80.0)
=12 years of schooling	880	62.6 (59.5-65.7)	64.3 (60.3-68.3)	68.8 (65.3-72.4)
>12 years of schooling	476	61.9 (53.6-70.3)	65.8 (58.5-73.1)	72.7 (68.5-76.9)
**Marital status**				
Never married	1,337	63.9 (59.2-68.6)	63.5 (57.9-69.0)	71.4 (67.2-75.5)
Separated, widowed, or divorced	489	66.6 (54.2-79.0)	64.5 (60.9-68.1)	70.9 (50.0-91.8)
Married	1,204	62.7 (55.8-69.6)	60.3 (56.2-64.4)	69.5 (54.8-84.3)
**Ethnicity**				
Black African	1,763	65.5 (62.8-68.2)	65.1 (59.7-70.6)	74.4 (70.6-78.2)
Colored	555	50.5 (43.8-57.2)	47.6 (33.4-61.9)	56.1 (41.9-70.3)
Indian or Asian	374	52.6 (49.8-55.4)	65.4 (60.7-70.0)	65.2 (60.5-69.8)
White	395	67.9 (67.4-68.4)	59.0 (58.8-59.3)	61.5 (60.3-62.8)
**Residence type**				
Urban	2,246	62.9 (60.9-64.9)	63.1 (60.2-66.0)	69.0 (69.0-69.1)
Rural	866	65.8 (61.0-70.5)	61.5 (58.7-64.3)	73.7 (68.1-79.2)
**Current Smoking of combustible tobacco products**^**a**^				
Non smoker	2,410	70.5 (68.3-72.8)	71.3 (63.0-79.5)	78.4 (76.8-80.0)
Current smoker	633	36.1 (25.7-46.5)	25.9 (22.4-29.5)	37.7 (31.8-43.6)

Note: *CI* = confidence interval. Denominators for the various environments assessed included only participants whose responses indicated that they worked, had a home, or had a car in their household respectively. The proportion of missing/inapplicable responses excluded from the denominators for the respective environments included: work (17.6%; n = 559); home (3.2%; n = 117); and car (16.4%; n = 458).

^a^Current smokers were respondents who reported daily or some days smoking of manufactured or hand-rolled cigarettes, cigars, pipes, or water-pipes.

There were no significant differences in the prevalence of 100% smoke-free policies in the home, workplace or private cars when stratified by age, sex, education level, residence type or marital status. However, significant within-group differences were observed by ethnicity for 100% smoke-free policies in all the environments assessed. During 2010, presence of 100% smoke-free policies in work-place was highest among Whites (67.9%) and lowest among Colored respondents (50.5%). Presence of 100% smoke-free policies in the home was highest among Indian or Asian respondents in 2010 (65.4%) and lowest among Colored respondents (47.6%). The proportion of South African adults that had 100% smoke-free policies in their private cars in 2010 was highest among black Africans (74.4%) and lowest among Colored respondents (56.1%).

Overall, the vast majority of nonsmoking South African adults perceived that smoke-free environments were important (Table 2), although this perceived importance was significantly lower for hospitality venues such as cafes/restaurants (86.1%) or at shabeens, bars or clubs (66.2%), compared to at work (91.2%), home (93.1%), or in hospitals (94.7%) (Table 2). Virtually all (99.6%) non-smokers believed that SHS was harmful.

**Table 2:** **Proportion of non-smoking **^***a ***^**South African Adults aged ≥ 16 years that reported that 100**% **smoke-free environments in private and public places were important to them, South African Social Attitudes Survey, 2010 (n = 2,410)**

**Characteristics**	**Sample**	**Work**	**Home**	**Hospitals**	**Café/Restaurants**	**Shabeens (local bars), Bars or Clubs**
		**% (95% CI)**	**% (95% CI)**	**% (95% CI)**	**% (95% CI)**	**% (95% CI)**
**Overall**	2,410	91.2 (87.2-95.1)	93.1 (89.1-97.0)	94.7 (92.4-97.0)	86.1 (83.3-88.9)	66.2 (63.2-69.2)
**Sex**						
Male	1,353	91.1 (87.4-94.8)	92.9 (88.0-97.9)	94.5 (92.1-96.9)	85.1 (81.6-88.5)	65.6 (63.0-68.2)
Female	1,044	91.4 (84.8-98.0)	93.5 (89.7-97.3)	95.2 (92.5-97.9)	87.6 (84.1-91.1)	67.8 (63.1-72.4)
**Age, years**						
16-24	496	91.6 (85.9-97.3)	92.5 (88.8-96.2)	94.7 (92.1-97.3)	81.7 (76.2-87.2)	59.9 (56.6-63.1)
25-34	536	89.3 (77.5-100.0)	92.2 (84.6-99.7)	92.9 (86.2-99.7)	85.8 (81.5-90.1)	66.9 (61.3-72.6)
35-44	499	92.3 (87.8-96.7)	94.1 (88–100.0)	96.6 (94.8-98.4)	87.2 (82.2-92.2)	64.2 (57.7-70.6)
45-54	339	92.9 (89.6-96.1)	96.0 (92.0-100.0)	97.5 (96.1-98.8)	90.4 (85.8-95.1)	71.7 (62.3-81.1)
≥55	539	91.0 (82.9-99.0)	92.1 (86.3-97.9)	93.4 (87.9-98.9)	89.2 (83.6-94.8)	73.4 (70.6-76.2)
**Education**						
<12 years of schooling	1,322	90.9 (86.5-95.3)	92.6 (87.5-97.7)	94.5 (91.5-97.4)	86.4 (84.1-88.7)	66.5 (62.2-70.9)
=12 years of schooling	695	92.3 (85.6-99.0)	93.2 (89.0-97.5)	94.9 (89.8-100.0)	85.3 (81.3-89.4)	67.5 (62.0-73.0)
>12 years of schooling	362	89.4 (85.1-93.8)	94.4 (93.3-95.5)	94.9 (93.6-96.1)	86.2 (73.9-98.5)	60.5 (44.2-76.7)
**Marital status**						
Never married	1,069	91.4 (83.4-99.4)	93.1 (88.4-97.8)	95.1 (92.6-97.7)	84.8 (82.0-87.6)	63.0 (59.4-66.7)
Separated, widowed, or divorced	384	91.2 (83.2-99.2)	93.5 (86.6-100.0)	93.2 (86.2-100.0)	86.4 (82.3-90.5)	66.7 (56.6-76.7)
Married	920	90.5 (82.5-98.4)	92.6 (87.7-97.5)	94.3 (90.6-98.0)	87.8 (81.7-93.9)	70.7 (66.2-75.2)
**Ethnicity**						
Black African	1,484	91.0 (85.8-96.2)	93.3 (89.7-97.0)	94.1 (90.4-97.7)	86.3 (83.6-89.1)	65.5 (60.8-70.1)
Colored	349	89.6 (78.5-100.0)	89.6 (61.0-100.0)	97.2 (95.6-98.8)	84.0 (63.4-100.0)	69.3 (50.6-87.9)
Indian or Asian	294	95.1 (93.8-96.4)	95.6 (94.4-96.7)	97.4 (96.7-98.1)	88.3 (85.3-91.4)	65.9 (60.4-71.4)
White	264	93.3 (92.9-93.8)	93.5 (93.1-94.0)	98.5 (98.4-98.6)	85.2 (84.2-86.3)	73.0 (71.2-74.9)
**Residence type**						
Urban	1,689	90.2 (88.5-91.9)	93.0 (92.1-93.9)	94.3 (92.4-96.2)	87.1 (86.8-87.4)	67.1 (62.0-72.1)
Rural	721	92.7 (87.0-98.5)	93.2 (83.1-100.0)	95.3 (91.4-99.3)	84.4 (79.3-89.5)	64.7 (60.5-69.0)

Note: *CI* = confidence interval.

^a^Non-smokers were respondents who reported not smoking hand-rolled or manufactured cigarettes, cigars, pipes, or water-pipes.

### Exposure to SHS at home, in the workplace and at hospitality venues among non-smokers

Overall, 55.9% of all non-smoking South African adults reported exposure to SHS from at least one source (i.e., in the home, workplace, café/restaurant or at a shabeen, bar, or club) during 2010. By specific source of exposure, 18.4% reported being exposed to SHS at work, 25.2% at home, 33.4% in a café/restaurant, and 32.7% at a shabeen, bar, or club (Table 3).

**Table 3:** **Prevalence of Secondhand Smoke Exposure in different environments among nonsmoking **^***a***^**South African Adults aged ≥ 16 years, South African Social Attitudes Survey 2010 (n = 2,410)**

**Characteristic**	**Sample**	**Work**	**Home**	**Café or Restaurants**	**Shabeens (local bars), Bars or Clubs**	**Any exposure**^***b***^
		**% (95% CI)**	**% (95% CI)**	**% (95% CI)**	**% (95% CI)**	**% (95% CI)**
**Overall**	2,410	18.4 (15.7-21.2)	25.2 (15.7-34.6)	33.4 (25.4-41.4)	32.7 (25.7-39.8)	55.9 (52.3-59.4)
**Sex**						
Male	1,353	20.7 (19.4-22.0)	27.0 (19.4-34.7)	34.7 (28.9-40.5)	34.2 (27.7-40.7)	58.2 (54.1-62.3)
Female	1,044	15.1 (10.6-19.5)	22.5 (8.4-36.5)	31.3 (21.3-41.3)	30.3 (19.6-40.9)	52.7 (47.8-57.6)
**Age, years**						
16-24	496	11.1 (0.9-21.3)	25.3 (19.8-30.9)	34.7 (25.8-43.6)	36.3 (19.8-52.9)	60.5 (54.5-66.6)
25-34	536	26.0 (23.0-29.1)	31.2 (13.6-48.9)	36.2 (31.8-40.5)	44.2 (28.9-59.5)	62.0 (55.6-68.4)
35-44	499	24.4 (14.3-34.6)	23.2 (8.0-38.4)	32.6 (24.5-40.7)	27.7 (22.7-32.7)	52.7 (46.3-59.2)
45-54	339	20.2 (11.2-29.1)	22.0 (18.0-25.9)	35.5 (15.3-55.7)	26.3 (13.7-38.8)	53.6 (46.0-61.1)
≥55	539	10.8 (7.7-13.9)	20.0 (3.3-36.7)	26.6 (12.0-41.2)	19.4 (15.0-23.8)	43.9 (36.8-51.0)
**Education**						
<12 years of schooling	1,322	14.1 (7.9-20.2)	28.0 (21.0-34.9)	28.1 (23.7-32.6)	28.5 (20.7-36.2)	53.0 (48.3-57.7)
=12 years of schooling	695	23.1 (14.2-31.9)	24.8 (10.2-39.4)	39.4 (36.9-42.0)	38.4 (28.0-48.8)	61.2 (55.4-67.1)
>12 years of schooling	362	25.4 (19.7-31.0)	15.6 (13.5-17.7)	40.8 (22.6-59.1)	37.3 (27.7-46.9)	55.7 (48.7-62.6)
**Marital status**						
Never married	1,069	17.2 (15.3-19.1)	26.7 (16.6-36.8)	33.4 (30.0-36.7)	36.0 (27.9-44.1)	58.1 (53.5-62.7)
Separated, widowed, or divorced	384	13.8 (0.0-29.8)	23.1 (8.3-38.0)	29.2 (7.2-51.2)	24.2 (17.8-30.6)	46.3 (40.2-56.4)
Married	920	22.5 (19.8-25.2)	24.1 (14.3-33.9)	34.8 (22.6-47.0)	29.5 (20.6-38.3)	54.3 (49.3-59.2)
**Ethnicity**						
Black African	1,484	18.2 (15.2-21.2)	26.3 (17.7-34.9)	32.0 (26.7-37.4)	34.0 (23.7-44.3)	55.8 (51.6-60.1)
Colored	349	23.4 (7.5-39.3)	30.1 (17.1-43.1)	33.4 (29.5-37.3)	23.4 (15.1-31.7)	56.5 (48.5-64.6)
Indian or Asian	294	19.8 (15.4-24.1)	13.0 (10.7-15.2)	36.7 (30.2-43.1)	24.8 (20.5-29.2)	55.3 (46.4-64.1)
White	264	16.7 (15.4-17.9)	15.7 (14.5-16.8)	44.8 (43.1-46.5)	28.3 (26.2-30.5)	55.6 (48.3-62.8)
**Residence type**						
Urban	1,689	18.9 (16.6-21.2)	23.3 (13.0-33.6)	35.9 (31.6-40.2)	35.2 (27.2-43.1)	57.3 (53.4-61.3)
Rural	721	17.7 (15.0-20.4)	28.2 (27.7-28.7)	29.4 (27.6-31.1)	28.8 (26.6-31.1)	53.4 (46.6-60.3)

Note: *CI* = confidence interval. Denominators for the various environments assessed included only participants whose responses indicated that they worked, had a home, had a car in their household, or that they went to cafes/restaurants; or to shabeens, bars or clubs, respectively. The proportion of missing/inapplicable responses excluded from the denominators for the respective environments included: work (16.1%; n = 380); home (2.9%; n = 69); cafes/restaurants (6.3%; n = 134); and shabeens, bars or clubs (9.8%; n = 254),

^a^Non-smokers were respondents who reported not smoking hand-rolled or manufactured cigarettes, cigars, pipes, or water-pipes.

^b^Respondents who reported being exposed to secondhand smoke in at least one of the following: the work, home, café/restaurants, or bars, shabeens, or clubs.

After adjusting for all other factors, females had significantly lower odds of being exposed to SHS at work (*a*OR = 0.81; 95% CI: 0.75-0.88) and at home (*a*OR = 0.73; 95% CI: 0.620.87) but did not differ significantly from males with respect to SHS at café/restaurants and at shabeens, bars, or clubs (Table 4). Compared to respondents aged 16–24 years, the odds of SHS exposure in a shabeen, bar, or club were significantly lower among older respondents aged 45–54 years (*a*OR = 0.47; 95% CI: 0.38-0.60). Similarly, respondents aged ≥55 years had lower odds of being exposed to SHS in a café/restaurant compared to those aged 16–24 years (*a*OR = 0.59; 95% CI: 0.42-0.82).

**Table 4:** **Adjusted correlates of secondhand smoke exposure in different environments among nonsmoking **^***a ***^**South African Adults aged ≥ 16 years, South African Social Attitudes Survey 2010**

**Characteristics**	**Category**	**Work**	**Home**	**Café/Restaurant**	**Shabeen, (local bars), Bar or Club**	**Any secondhand smoke exposure**^***b***^
		**aOR (95% CI)**	**aOR (95% CI)**	**aOR (95% CI)**	**aOR (95% CI)**	**aOR (95% CI)**
**Sex**	Male (Referent)					
	Female	0.81 (0.75-0.88)*	0.73 (0.62-0.87)*	0.95 (0.72-1.25)	0.78 (0.47-1.28)	0.74 (0.63-0.87)*
**Age, years**	16-24 (Referent)					
	25-34	2.23 (0.59-8.47)	1.43 (0.56-3.67)	1.02 (0.56-1.85)	1.04 (0.37-2.92)	1.18 (0.62-2.24)
	35-44	2.06 (0.39-10.82)	0.83 (0.23-2.94)	0.73 (0.41-1.29)	0.51 (0.17-1.49)	0.77 (0.35-1.66)
	45-54	1.72 (0.52-5.65)	0.77 (0.26-2.27)	0.87 (0.72-1.05)	0.47 (0.38-0.6)*	0.79 (0.55-1.12)
	≥55	0.88 (0.21-3.79)	0.66 (0.15-2.85)	0.59 (0.42-0.82)*	0.34 (0.1-1.21)	0.56 (0.17-1.79)
**Education**	<12 years of schooling (Referent)					
	=12 years of schooling	1.60 (0.64-3.97)	0.67 (0.34-1.31)	1.37 (1.07-1.77)*	1.29 (0.94-1.76)	1.33 (1.06-1.67)*
	>12 years of schooling	1.63 (1.13-2.37)*	0.41 (0.27-0.63)*	1.16 (0.49-2.79)	1.23 (0.67-2.23)	1.22 (0.68-2.2)
**Marital status**	Never married (Referent)					
	Separated, widowed, or divorced	0.93 (0.24-3.70)	1.21 (0.77-1.89)	1.20 (0.96-1.49)	1.08 (1.02-1.14)*	1.12 (0.51-2.44)
	Married	1.22 (1.03-1.45)*	0.98 (0.45-2.16)	1.14 (0.91-1.42)	1.13 (0.75-1.69)	1.13 (0.93-1.36)
**Ethnicity**	Black African (Referent)					
	Colored	1.45 (0.60-3.52)	1.53 (0.91-2.55)	1.10 (0.97-1.25)	0.57 (0.34-0.96)*	0.85 (0.49-1.48)
	Indian or Asian	0.78 (0.52-1.17)	0.85 (0.44-1.64)	0.75 (0.58-0.98)*	0.4 (0.24-0.65)*	0.85 (0.68-1.06)
	White	0.78 (0.65-0.94)*	0.84 (0.41-1.71)	1.42 (1.08-1.87)*	0.65 (0.42-1)	0.8 (0.62-1.04)
**Residence type**	Urban (Referent)					
	Rural	1.08 (0.80-1.46)	1.16 (0.78-1.73)	0.76 (0.71-0.80)*	0.62 (0.47-0.81)*	0.79 (0.65-0.95)*

Note: Denominators for the various environments assessed included only participants whose responses indicated that they worked, had a home, had a car in their household, or that they went to cafes/restaurants; or to shabeens, bars or clubs, respectively. The proportion of missing/inapplicable responses excluded from the denominators for the respective environments included: work (16.1%; n = 380); home (2.9%; n = 69); cafes/restaurants (6.3%; n = 134); and shabeens, bars or clubs (9.8%; n = 254),

^a^Non-smokers were respondents who reported not smoking hand-rolled or manufactured cigarettes, cigars, pipes, or water-pipes. *aOR* = adjusted odds ratio; *CI* = confidence interval.

^b^Respondents who reported being exposed to secondhand smoke in at least one of the following: the work, home, restaurants, or bars.

*Statistically significant at *p* < 0.05.

By education, respondents with >12 years of secular education had lower odds of being exposed to SHS at home (*a*OR = 0.41; 95% CI: 0.27-0.63) but higher odds of being exposed to SHS at work (*a*OR = 1.63; 95% CI: 1.13-2.37) compared to those with <12 years of education. Also, those with 12 years of education had higher odds of being exposed to SHS at a café/restaurant compared to those with <12 years of education (*a*OR = 1.37; 95% CI: 1.07-1.77).

By marital status, respondents who were married had higher odds of being exposed to SHS at work compared to those were never married (*a*OR = 1.22; 95% CI: 1.03-1.45). Also, the odds of SHS exposure in a shabeen, bar, or club were significantly higher among respondents who were separated, widowed or divorced compared to those who were never married (*a*OR = 1.08; 95% CI: 1.02-1.14).

Whites had lower odds of being exposed to SHS at work compared to black Africans (*a*OR = 0.78; 95% CI: 0.65-0.94), but higher odds of being exposed to SHS at a café/restaurant compared to black Africans (*a*OR = 1.42; 95% CI: 1.08-1.87), Also, the odds of SHS exposure in a shabeen, bar, or club were significantly lower among Indians/Asians (*a*OR = 0.40; 95% CI: 0.24-0.65) as well as among Coloreds (*a*OR = 0.57; 95% CI: 0.34-0.96) when compared to black Africans.

### Effect of smoking bans in protecting non-smokers from SHS smoke

Presence of work bans on indoor smoking conferred lower odds of exposure to SHS at work (*a*OR = 0.23; 95% CI: 0.09-0.60). Similarly, presence of 100% smoke-free home rules decreased the odds of being exposed to SHS at home (*a*OR = 0.16; 95% CI: 0.09-0.30).

## Discussion

The findings from this study showed that during 2010, about two-thirds of the adult population had 100% smoke-free policies in their homes or workplaces, while approximately 3 of every 5 had adopted smoke-free rules in their private cars. These findings are a clear indication that the majority of South African adults are not only aware of the harmful health effects of involuntary exposure to SHS, but are also taking positive actions to protect themselves from such exposure. However, our findings showed that about half (55.9%) of non-smokers were still exposed to SHS from at least one source. This underscores the need for total bans on indoor smoking in public places with no exemptions. Such 100% smoke-free laws would not only be simpler and more consistent to enforce, but would also be more effective in reducing SHS exposure, particularly among individuals who currently work in designated smoking areas at hospitality venues. This is particularly important because, many nonsmoking employees who work in such areas (generally individuals of low socio-economic status), may be afraid to assert their right to smoke-free air at their work place because of fear of upsetting their employers [3].

The fact that virtually all non-smokers believed that SHS exposure was harmful and the vast majority indicated their support for 100% smoke-free policies in private and public areas could have some policy implications. For example, it may underscore the need for population-based educational campaigns which provide smoking cessation advice, or information on how to help a smoking friend or relative to quit. Such campaigns could also be opportunities to encourage non-smokers to be active citizens in enforcing smoke-free laws, e.g., by calling the appropriate enforcement agency in instances of violations of indoor-smoke free laws. Such concerted efforts by both individual non-smokers, and law enforcement officers may help denormalize smoking in public areas, thus encouraging smoke-free environments.

Our findings also showed some disparities in exposure to SHS among population subgroups. For example, women were significantly more likely than men to work in places with 100% smoke-free policies, which has been observed in previous research [10]. This may be due to the higher prevalence of females in certain professions where smoke-free environments are especially the norm, including nurses, child-minding, clerical staff and school teachers. There is also evidence that white-collar workers are twice as likely to be covered by smoke-free policies as blue-collar workers and women form a higher proportion of the population of white-collar workers compared to blue-collar workers [10,11]. The fact that the majority of those who self-identified as Coloreds were permissive of smoking in their homes may be related to the fact that this population group also has the highest smoking prevalence (36.3% vs. national average of 18.1%) in South Africa, and smoking may thus have become a norm. Similarly, the fact that the prevalence of smoke-free policies was consistently higher among non-smokers than smokers in all environments could be a result of the tendency of smokers to continue smoking in such environments with only partial bans on indoor smoking, which could undermine efforts to denormalize smoking [12-14].

The prevalence of exposure to SHS at hospitality venues during 2010 among non-smokers was higher than exposure at workplaces, homes and cars. The proportion of South African non-smokers who were exposed to SHS at a café/restaurant (33.4%) was similar to exposure at a bar, shabeen (local bar) or nightclub (32.7%). This may reflect the laxity in the implementation of smoke-free policies in such venues possibly due to commercial interests of business owners and less government involvement in the enforcement of these polices in such locations. This underscores the need for enhanced and sustained efforts to further reduce SHS exposure among all sub-population groups through stronger enforcement of smoke-free policies coupled with intensified population-based interventions aimed at reducing smoking prevalence and intensity among current smokers.

This study is the first to assess the implementation of smoke-free policies at work-places, and voluntary adoption of smoke-free rules in private environments such as the home and in cars, using a nationally representative sample of South African adults. Nonetheless, the study has some limitations. First, SHS exposure was self-reported and may have been subject to misreporting. However, recall was limited to the past 30 days, which is a relatively short period. Second, it is possible for respondents to have misclassified the presence of smoke-free rules in their homes or cars (e.g., indicating they had 100% smoke-free rules when such rules did not exist), possibly because of perceived social desirability of the provided responses. Finally, these data may not be generalizable to military or other institutionalized personnel who were not included in the survey, and who may have higher smoking prevalence rates. Despite these limitations, this study underscores the need for comprehensive smoke-free laws in public places to protect non-smoking adults and children from involuntary exposure to SHS.

## Conclusion

Despite the fact that the vast majority of non-smoking South African adults knew about the harmfulness of SHS exposure, with over two-thirds having implemented smoke-free rules in their cars and homes, over half still reported SHS exposure from several sources, particularly from public areas. This underscores the need for comprehensive smoke-free laws that prohibit smoking in all indoor areas without exemptions. In addition, strong enforcement of such laws may help increase compliance and denormalize smoking.

## Competing interests

The authors declare that they have no competing interests.

## Authors’ contributions

OA-AY was involved in acquisition of the data used for the study, conceiving the study design, interpreting the results of the analysis, and making substantial contributions to the drafting of the manuscript. OO searched relevant literature, made substantial contributions to the presentation of the data, and participated in drafting the final manuscript. ITA contributed to the design of the study, carried out the analysis of the data, and drafted the initial manuscript. All authors read and approved the final manuscript.

## Authors’ information

OAA is a public health practitioner and holds the position of Professor and Director, at the School of Oral Health Sciences at the University of Limpopo Medunsa Campus. OAA is also an Extraordinary Professor in the School of Health Systems and Public Health at the University of Pretoria. OO is MPH student at the Harvard School of Public Health. ITA initiated the reported research while affiliated with the Center for Global Tobacco Control at Harvard University. He is currently affiliated with the Centers for Disease Control and Prevention’s Office on Smoking and Health. The research in this report was completed and submitted outside of the official duties of his current position and does not reflect the official policies or positions of the Centers for Disease Control and Prevention.

## Pre-publication history

The pre-publication history for this paper can be accessed here:

http://www.biomedcentral.com/1471-2458/14/580/prepub
